# Assessment of Axial Behavior of Circular HPFRCC Members Externally Confined with FRP Sheets

**DOI:** 10.3390/polym10020138

**Published:** 2018-01-31

**Authors:** Ugur Demir, Yusuf Sahinkaya, Medine Ispir, Alper Ilki

**Affiliations:** Civil Engineering Faculty, Istanbul Technical University, Maslak, Istanbul 34469, Turkey; sahinkayayusuf@itu.edu.tr (Y.S.); ispirm@itu.edu.tr (M.I.); ailki@itu.edu.tr (A.I.)

**Keywords:** concrete, confinement, ductility, HPFRCC, FRP

## Abstract

The aim of this paper is to identify the axial behavior characteristics of FRP (fiber reinforced polymer) confined circular HPFRCC (high performance fiber reinforced cementitious composite) members under compression. The test program comprised of 24 circular specimens with an average compressive strength of 102.7 MPa, including 21 carbon FRP (CFRP) confined (2, 4, 6, 8 and 10 layers) and three unconfined specimens. Transverse confinement generated by external FRP sheets resulted with a remarkable enhancement in axial strength and deformability, which is extremely important to resist seismic actions. The higher was the thickness of FRP confinement, the larger was the ultimate strain (ε_cu_) and peak compressive strength (f′_cc_) of externally confined HPFRCC. When compared to FRP confined conventional concrete, different axial and lateral deformation characteristics were seen in FRP jacketed HPFRCC members. Higher strength and steel fiber presence in HPFRCC limited the lateral deformations which resulted with reduced strain efficiency with respect to conventional concrete. After presenting the experimental work, performance and accuracy of several available models proposed for predicting the axial behavior of FRP jacketed concrete were evaluated in a comparative manner.

## 1. Introduction

Decreasing the dimensions and self-weights of reinforced concrete (RC) structural members leads to feasible architectural solutions as well as reduced seismic forces. For this purpose, use of high strength concrete (HSC) in the construction industry has been a promising approach. However, increasing the strength of concrete generally causes brittleness which should be avoided in design of ductile reinforced concrete structures. To eliminate this drawback in higher strength concretes, one solution is adding steel fibers for achieving a more ductile behavior through keeping crack width low and thus increasing energy absorption capability of RC members. On the other hand, it has also been reported that the smaller is the aggregate size, the higher is the load carrying and impact resistance, since bond strength between cement and aggregate is higher when the aggregate size is smaller [1]. These types of composites including smaller size aggregate and fiber reinforcement are classified as high-performance fiber reinforced cement composite (HPFRCC). HPFRCCs perform large strain hardening upon peak stress resulting with high tensile and compressive strengths with respect to conventional steel fiber reinforced concrete [2]. Nowadays, such materials are used for high-rise buildings, long-span bridges, highways, and airfield pavements [3]. Therefore, safety and reliability of this type of composites should be adequately ensured. The most remarkable disadvantage of HPFRCC is the high cost of the material associated with fibers, silica fume, significantly higher amount of cement, etc. Currently HPFRCC is only used for critical applications where use of conventional concrete does not warrant the specific requirements of the project. The cost of this innovative material is expected to be reduced to a competitive level in the future with further research and resulting more widespread use of the material.

Since the behavior under compression is more brittle in HPFRCC compared to normal strength concrete (NSC), FRP materials can be alternatively used to prevent brittle failure through lateral confinement. Although the existing studies published in the literature on the axial compressive behavior of FRP-confined concrete have extensively focused on NSC (e.g., [4,5,6,7,8,9,10]) and HSC (e.g., [11,12,13,14,15,16,17,18,19,20]), less is known about the axial behavior of FRP confined HPFRCC columns. Studies on FRP confined concrete including fiber reinforcement is rare in the literature. Xie and Ozbakkaloglu [19] presented the axial compressive behavior of steel fiber reinforced high strength concrete (SFRHSC) and slurry infiltrated fiber concrete (SIFCON) filled FRP tubes with circular cross-sections and Zohrevand and Mirmiran [20] tested ultra-high-performance concrete (UHPC) filled circular FRP tubes with unconfined compressive strength of 189 MPa. Specimens were confined by glass (GFRP) or carbon fiber reinforced polymer (CFRP) tubes. In Xie and Ozbakkaloglu [19], steel fibers were added at volume fractions of 1.5% or 2.5% for SFRHSC specimens and 5% for SIFCON specimen while in Zohrevand and Mirmiran [20], a metallic fiber content of 2% in volume was used. 

According to the best knowledge of the authors, this paper summarizes the axial behavior of FRP jacketed high performance steel fiber reinforced cementitious composites for the first time in the literature. The originality of the presented research is external confinement of steel fiber reinforced cementitious composites without coarse aggregates, not typical concrete. All previous studies have focused on confinement of normal or high strength concrete (e.g., [4,5,6,7,8,9,10,11,12,13,14,15,16,17,18,19,20]). It is also worth mentioning that Xie and Ozbakkaloglu [19] and Zohrevand and Mirmiran [20] tested concrete members with steel fibers. However, Xie and Ozbakkaloglu [19] have focused on HSC and SIFCON filled FRP tubes and Zohrevand and Mirmiran [20] have studied UHPC filled FRP tubes. None of these studies have considered external jacketing with FRPs. In the light of the literature review, the axial behavior of HPFRCC specimens with circular cross sections externally confined by 2, 4, 6, 8 and 10 layers of FRP jackets was investigated through testing for the first time. It was observed that FRP confinement provides significant enhancement in terms of ultimate strain (ε_cu_) and compressive strength (f′_cc_) of HPFRCC. Available analytical models [21,22,23,24,25,26] applicable to FRP confined conventional (NSC, HSC) concrete are also assessed in terms of predicting the axial behavior of HPFRCC specimens jacketed with FRP. Although the model proposed by Lim and Ozbakkaloglu [26] gave satisfactory results in predicting the ultimate strain and axial strength of HPFRCC, there is a need for further experimental and analytical works to develop a better model which can realistically consider the sudden strength drop between the first and the second peak of the stress strain curves which is discussed in detail in the following sections. 

## 2. Materials and Methods

A total of 24 HPFRCC specimens with circular cross-sections were produced by a local concrete supplier (ISTON) with a diameter of 150 mm and a height of 300 mm. The test program comprised of 21 FRP confined and 3 unconfined specimens where CFRP sheets were used for external confinement. Average unconfined compressive strength was obtained as 102.7 MPa and the corresponding average axial strains at peak stress were measured as 0.0032. Description of the test specimens are summarized in Table 1. For instance, CC-C-6 is the specimen that was made of HPFRCC, with circular cross-section, and confined by six layers of CFRP. The volumetric ratios of FRP (ρ_f_) given in Table 1 are calculated using Equation (1) as recommended by [4]. In Equation (1), t_f_ is the total effective thickness of FRP and D is the diameter of the specimens.

(1) $$\rho_{f}=\frac{4t_{f}}{D}$$

**Figure 1:**
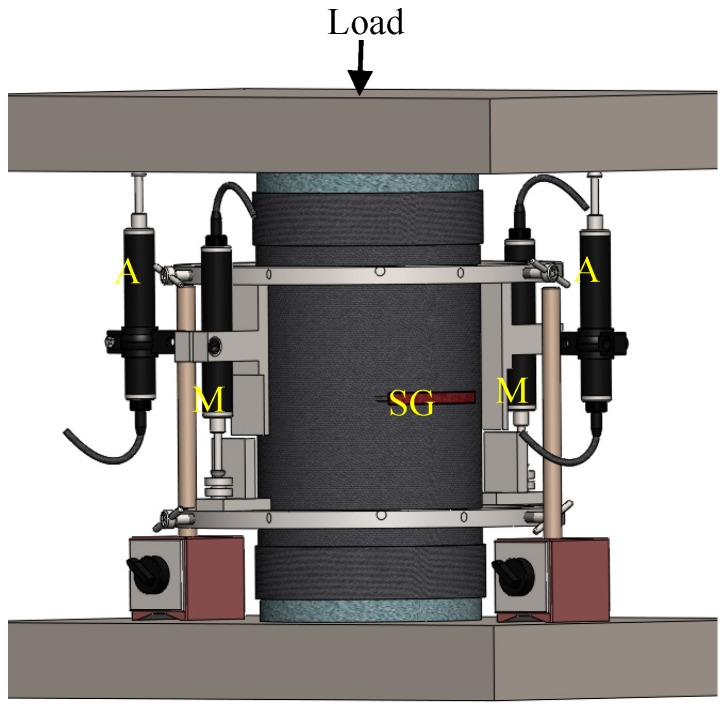
Test setup illustrating LVDT and strain gauge applications (M: Mid-height LVDT; A: All-height LVDT; SG: Strain gauge).

Mix proportions of HPFRCC are given in Table 2. Maximum aggregate size of sand was 0.5 mm and no coarse aggregate was used. It should be clarified that this research deals with confinement of a high performance cementitious composite and not a concrete, hence there is no coarse aggregate in the concrete mix. Steel fibers with a diameter of 0.9 mm and length of 60 mm, referring to an aspect ratio (length/diameter) of 67 were used in the composite mix. The steel fiber volume fraction *V_f_* (volume of fibers per unit volume) was 1%. Mechanical properties of unidirectional CFRP sheets provided by the manufacturer are given in Table 3. The epoxy used for bonding CFRP sheets to composite consisted of a resin binder and hardener, which are mixed, in a ratio of 2/1 by weight. Density and viscosity of the epoxy-binder mix given by manufacturer are 1.05 kg/L and 500 cp, respectively. The surface of the specimens was cleaned before the epoxy was applied. A thin layer of epoxy primer was then applied to surface of the specimens to fill voids. Afterwards, the two-compound epoxy system was hand mixed according to the manufacturer’s provisions and applied to the surface of the specimens. The impregnated CFRP sheets were then wrapped around the specimens with fibers oriented in hoop direction. An overlap of 150 mm was formed at the end of the wrap to ensure the development of composite strength in the outermost external layer. Compression tests were carried out under monotonic uniaxial compressive loading by using an Instron testing machine with a capacity of 5000 kN. The tests were displacement controlled with a loading rate of 0.5 mm per minute. For measuring axial deformations, two, linear variable differential transducers (LVDTs) were located at the mid height of the specimen with a gauge length of 150 mm (Figure 1). Additionally, two LVDTs were placed between the loading and supporting steel plates along the height of the specimen as seen in Figure 1. To measure the lateral strains, two strain gauges were placed at the mid-height of the specimen along the transverse direction. The gauge length of each strain gauge was 60 mm. A TML-TDS-303 data logger was used for data acquisition. Axial loads during the tests were taken from the built-in load cell of the testing machine. Axial stresses were computed by dividing the recorded loads to the initial cross section area of the specimen tested. The axial strains were calculated by dividing the average displacement readings of the LVDTs at mid-height to the gauge length of 150 mm.

## 3. Test Results

**Figure 2:**
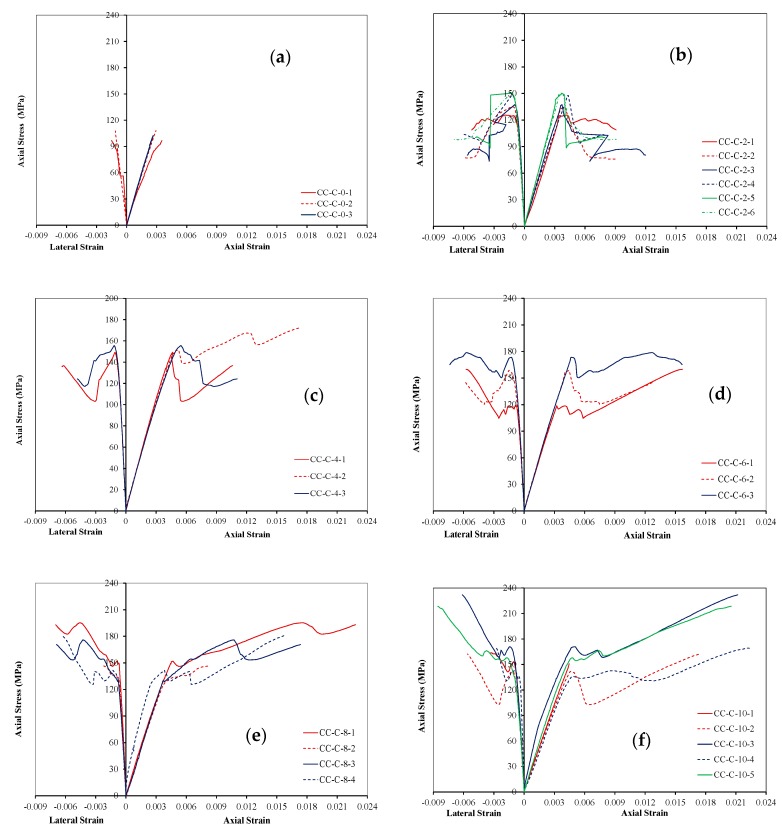
Axial stress–strain curves of HPFRCC specimens: (**a**) unconfined; (**b**) confined by two layers of CFRP; (**c**) confined by four layers of CFRP; (**d**) confined by six layers of CFRP; (**e**) confined by eight layers of CFRP; and (**f**) confined by ten layers of CFRP.

The axial behavior of circular HPFRCC specimens is presented with axial stress–strain relationships. These relationships are given in Figure 2 and numerical test results are presented in Table 4. In Figure 2, the negative parts of the horizontal axis are used to show lateral strains (ε_h_) whereas positive parts are used for axial strains (ε_c_). In Table 4, f′_co_ and ε_co_ are the unconfined axial strength and corresponding axial strain, whereas f′_cc_ and ε_cu_ are the compressive strength and ultimate axial strain of FRP confined specimens, respectively. f′_cu_ is the ultimate strength. It should be noted that, in this paper, the ultimate axial strain, which is the strain at sudden rupture of FRP, or the strain corresponding to 70% of the peak strength (whichever is less) is considered for confined specimens as ultimate point. On the other hand, axial strength was considered as the peak strength reached during the test. Stress–strain relationships of FRP confined HPFRCC specimens are different from those of FRP confined conventional concrete. Stress–strain behavior can be represented by a first ascending branch followed by a descending branch resulting with a sudden strength drop and in most cases with a second ascending branch. Although none of them were HPFRCC as the specimens tested in this study, similar behavior was also reported in FRP confined or tube encased variations of high/ultra-high strength/performance concrete specimens tested by Xie and Ozbakkaloglu [19] and Zohrevand and Mirmiran [20]. The descending and the second ascending branches are affected by the axial stiffness of FRP jacket. Higher the stiffness of FRP sheets, lower was the strength drop after the first peak of the stress–strain curves (Table 4). Moreover, initial modulus of elasticity (E_c_) is determined as the slope of the first ascending branch between 5% and 40% of the axial strength. As shown in Table 4 and as expected, average E_c_ values of confined HPFRCC specimens (38,356 MPa) are similar to those obtained for the unconfined HPFRCC specimens (34,266 MPa) whereas the slopes of the second ascending branches depend on the FRP stiffness. Distinctions on the transition between the first and the second ascending branches demonstrated the effectiveness of the FRP sheets. The effect of FRP confinement on deformability is much more pronounced than that on strength for HPFRCC. As seen in Table 4, for circular specimens confined with 2, 4, 6, 8 and 10 layers of CFRP, axial strengths were enhanced by 38%, 55%, 61%, 79% and 90%, respectively, while ultimate axial strains were improved by 110%, 332%, 391%, 521% and 580%, respectively. This enhancement is particularly important for HPFRCC members to be subjected to seismic actions. While the unconfined HPFRCC specimens fail in an extremely brittle manner as expected, the FRP jacketed HPFRCC specimens, when the stiffness of the jacket is sufficient, display a limited post-peak strength loss and then an ascending second branch occurs resulting with a more ductile behavior (Table 4). When the stiffness of the jacket was insufficient, the second peak in stress strain curves could not be observed or seen to be lower than the first peak. The sudden post-peak strength loss can be attributed to the lack of confinement effectiveness that could be eliminated using more amount of FRP volumetric ratio for slightly confined specimens. Xie and Ozbakkaloglu [19] stated that higher volume fraction of steel fibers (not less than 1.5%) can reduce the level of the strength loss right after the first peak for HSC.

The average value of the hoop rupture strain is obtained as about 0.006 for all specimens independent from FRP confinement ratio (Table 4). Using this average value, strain efficiency factor (k_ε_), which is the ratio of the hoop rupture strain to the ultimate uniaxial tensile strain of FRP, is calculated using Equation (2) as an average of 0.35. This value is lower with respect to FRP confined conventional concrete. For instance, Tamuzs et al. [21,22] reported the value of strain efficiency factor as 0.57 for FRP confined NSC and HSC with compression strengths up to 82 MPa. Similarly, Pellegrino and Modena [24] recommended the value of 0.50 for NSC and HSC, whereas, in the case of steel fiber reinforced high strength concrete filled FRP tubes, Xie and Ozbakkaloglu [19] obtained an experimental average value of 0.65.

The inverse relationship between unconfined concrete compressive strength and hoop rupture strain of FRP was also reported by other researchers (e.g., [12,15,16,17,18,19,20]). 

(2) $$k_{\varepsilon }=\frac{\varepsilon_{h,\mathrm{rup}}}{\varepsilon_{\mathrm{fu}}}$$

**Figure 3:**
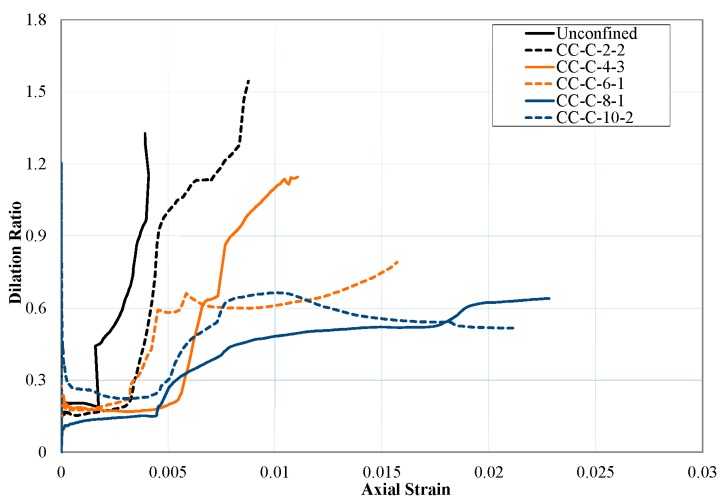
Dilation ratio-axial strain relationship in FRP confined HPFRCC.

The dilation ratio (μ) is calculated as the ratio of variations of lateral strain to axial strain (Equation (3)). As shown in Figure 3, the dilation ratio remained almost constant up to axial strains corresponding to peak strength of the unconfined HPFRCC. Upon reaching this point, a rapid increase was observed in dilation related with the sudden increase in lateral strains due to damaging of HPFRCC. The sudden drop of strength right after the first peak can also be explained with the sudden increase of dilation upon reaching of unconfined strength for the specimens which are not sufficiently confined (i.e., the specimens jacketed with two plies of FRP sheets). It can be seen in Figure 3 that, when stiffness of FRP jacket is higher, the increment in dilation at this point is less. Consequently, the sudden drop in axial strength is limited and the axial strength can be sustained until larger axial strains and higher deformability is achieved. For better confined specimens (i.e., specimens jacketed with 6, 8 or 10 plies of FRP sheets), dilation ratio remained almost constant until the end of tests at around the value of 0.6 after increasing suddenly to this value from the values of 0.15–0.20 at around axial strain levels of 0.003–0.005. It also worth mentioning that the characteristics of variation of dilation ratio with increasing axial strains are different with respect to that of conventional concrete reported by Ilki et al. [7] in terms of both numerical values and general shape.

(3) $$\mu_{i}=\frac{\varepsilon_{\mathrm{hi}}-\varepsilon_{\mathrm{hi}-1}}{\varepsilon_{\mathrm{ci}}-\varepsilon_{\mathrm{ci}-1}}$$

**Figure 4:**
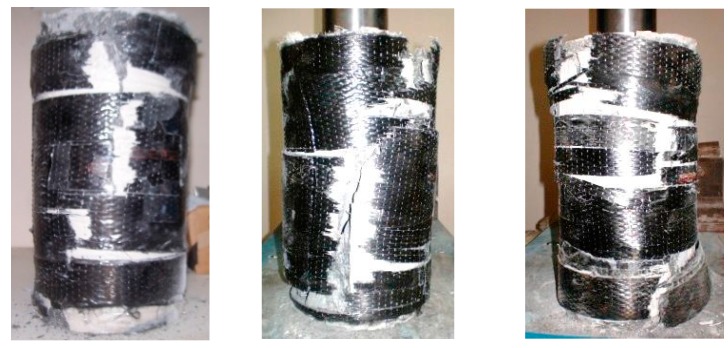
Typical failure modes.

Typical failure modes of specimens are shown in Figure 4. All specimens failed with an explosive rupture of the jackets. As seen in Figure 4, this failure mode was characterized with a major crack in HPFRCC along the height of the specimens. The specimens, which displayed almost identical failure modes, reached similar FRP hoop rupture strains independent of FRP thickness. The concentration of damage locally around the major cracks has probably caused local stress concentrations on the FRP jacket near the crack leading to lower measured FRP rupture strains. Similar local damages have also been reported for FRP confined HSC before [13,14,15,16,17,18,19,26]. 

## 4. Analytical Work

**Figure 5:**
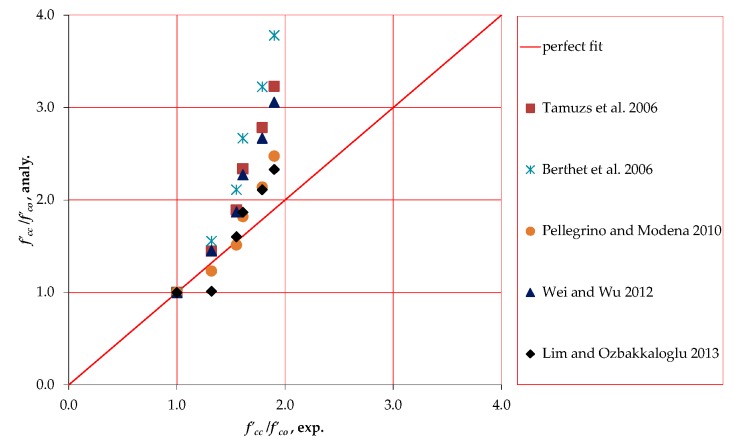
Axial strength predictions of investigated models.

**Figure 6:**
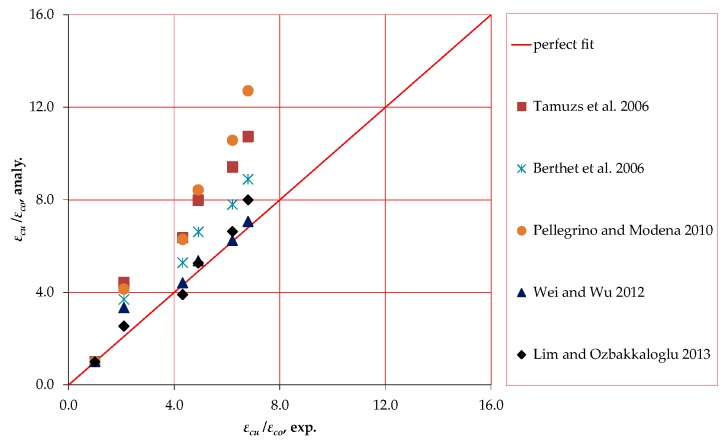
Ultimate axial strain predictions of investigated models.

No model in the published literature has been proposed specifically to predict the axial behavior of FRP confined circular HPFRCC. Therefore, five existing models [21,22,23,24,25,26], which have been proposed for FRP confined normal/high strength concrete, are used to predict the confined strength and ultimate strain of FRP confined HPFRCC specimens. The formulations proposed by these models for prediction of FRP confined strength and ultimate axial strain and the strength range that were considered during development of models are given in Table 5. The predictions of the models are plotted in Figure 5 and Figure 6 for axial strength and ultimate axial strain, respectively. It should be noted that, in Figure 5 and Figure 6, the horizontal and vertical axes refer to experimentally obtained and analytically predicted enhancement ratios, respectively. As seen in Figure 5 and Figure 6, out of the ordinary judgement for conventional concrete, majority of the investigated models could not predict the strength enhancements satisfactorily. Insufficient accuracy in predictions was more evident in case of increased FRP stiffness. Similarly, the higher was the stiffness of the FRP jackets, the larger was the scatter in terms of strain enhancement predictions. To evaluate the confined strength and ultimate strain predictions of the models statistically, the average absolute error (*AAE*) and standard deviation (*SD*) are calculated using Equations (4) and (5). In these equations, mod*_i_* and exp*_i_* indicate the predicted value by the model and the corresponding value determined by the test, respectively; mod*_avg_* and exp*_avg_* are the average values predicted by the model and determined by the test, respectively; and *n* is the number of tests. Table 6 presents the *AAE* and *SD* values calculated for each model. Wu and Zhou [27] defined the accuracy of a model based on *AAE* values; Category I (*AAE* ≤ 0.15), Category II (0.15 < *AAE* ≤ 0.30) and Category III (*AAE* > 0.30). The strength and ultimate strain predictions of the investigated models are categorized using this approach in Table 7.

(4) $$AAE=\frac{\sum_{i=1}^{n}|\frac{{\mathrm{mod}}_{i}-\exp_{i}}{\exp_{i}}|}{n}$$

(5) $$SD=\sqrt{\frac{\sum_{i=1}^{n}{\left(\frac{{\mathrm{mod}}_{i}}{\exp_{i}}-\frac{{\mathrm{mod}}_{avg}}{\exp_{avg}}\right)}^{2}}{n-1}}$$

As seen in Figure 5, and Table 6 and Table 7, the most accurate predictions in terms of axial strength were made by the model proposed by Pellegrino and Modena [24]. Only the model developed by Lim and Ozbakkaloglu [26] is placed in Category I in terms of both axial strength and strain prediction performances (Table 7). As seen in Table 6, Lim and Ozbakkaloglu [26] had relatively small *SD* values as well. Nevertheless, quite high scattering of predictions, particularly for well-confined sections, shows the need for further studies, not only for better prediction of axial strength and ultimate axial strain, but also for demonstrating the sudden drop and following hardening afterwards realistically (Figure 2), which will be addressed in the following section. Furthermore, the variation of other effective parameters, such as FRP type, jacket stiffness, unconfined axial strength, steel fiber strength/geometry/ratio and aspect ratio as well as other constituents of HPFRCC warrant the need for further research. It is worth clarifying that only the models proposed by Berthet et al. [23], Pellegrino and Modena [24] and Lim and Ozbakkaloglu [26] were established considering the compression strength range of specimens tested in the current study (102.7 MPa). However, these models were not directly developed for HPFRCC and focused on only FRP confined NSC and HSC behavior. Therefore, presented comparisons are only for checking whether these models can also be used for HPFRCC rather than suspecting their accuracies for what they originally were proposed for.

It should be noted that size effect does not significantly influence the axial behavior of FRP confined concrete and was not considered in this study. About this issue, Lorenzis et al. [28] reported that the size of CFRP-confined concrete cylinders had a weak influence on the compressive strength. Similarly, Théirault et al. [29] showed that conventional FRP-confined concrete cylinders can effectively be used to model the axial behavior of short columns whereas the size effect was clearly evident in only very small scale specimens (50 mm diameter). In addition, Carey and Harries [30] and Zhu et al. [31] observed that the size of FRP-confined concrete cylinders did not appear to have a significant effect on the confinement or the overall axial behavior. Furthermore, the current design codes (e.g., ACI 440.2R-08 [32], CSA-S806-2002 [33], and Turkish Seismic Design Code-2007 [34]) do not consider the size effect for concrete members strengthened with FRP either. 

## 5. Establishment of a Stress–Strain Model for HPFRCC

**Figure 7:**
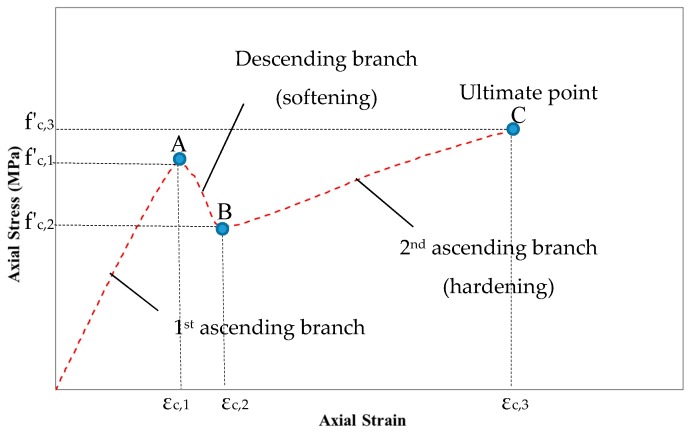
Three points (A, B and C) considered in modeling.

There is no model in the published literature, which has been proposed specifically to predict the axial behavior of FRP confined HPFRCC. Hence, based on the test data of this study, a model of axial stress-axial strain relationship is proposed. Considering the experimental stress-strain plots in Figure 2, the model is defined with three stress-strain points (A, B and C) and three linear parts connecting these points (Figure 7). It should be noted that this form of model considering the strength loss is proposed for the first time.

By performing regression analysis on the test data and using statistical parameters of the data such as mean, minimum and coefficient of variation, the stress and strain functions of these specific points are determined as explained in the following paragraphs:

*First point (Point A):* Since the lateral strain is low during the first ascending branch, there is no significant confinement effectiveness in this region. However, there are still increases in stress and strain with respect to the unconfined situation, as the wrapping allows the integrity of the specimen to be conserved and allow further redistribution of stresses preventing evolution of local and early damages. The fibrous nature of the HPFRCC, and the potential non-uniform distribution of the fibers can lead to a more heterogeneous structure than ordinary concrete resulting with a scatter in the first axial strength and corresponding strains. Therefore, the mean and coefficient of variation values could be evaluated rather than employing a regression analysis for the point A. It should be stated that this scatter (with conservative consideration in the model) does not cause any significant problem, just because the axial strength has already been preserved and lost for a very short period. The stress at this point varies from 1.15 to 1.74 with corresponding mean and coefficient of variation of 1.42 and 0.10, respectively, where the strain is in the range of 1.23–1.83 with corresponding mean and coefficient of variation of 1.47 and 0.12, respectively. In this study, the stress and strain at point A are recommended as the minimum values obtained experimentally to remain on conservative side. Therefore, stress and strain can be determined by Equations (6) and (7), respectively.

f_c,1_ = 1.15f_co_ (6)

ε_c,1_ = 1.20ε_co_ (7)

*Second point (Point B)*: When the first crack in HPFRCC occurs, the confinement pressure is low and the stress drop falls to a certain level where the specimen can solely carry. The stress at this point can be obtained by regression analysis and defined as in Equation (8). However, experimentally obtained strain values do not depend on the FRP thickness and, therefore, mean of experimental values can be considered in this case as given in Equation (9). *AAE* values for the stress and strain for point B are given in Table 8. The standard deviation of these values is calculated as 0.007.

f_c,2_ = f_co_ + 0.38f′_l_ (8)

ε_c,2_ =2.1ε_co_ (9)

*Third point (Point C)*: Point C is the ultimate point. Regression analysis is performed using experimental stress and strain values of this point and to predict these values, and Equations (10) and (11) are obtained.

f_c,3_ = f_co_ + f′_l_ (10)

ε_c,3_ = ε_co_ + 6ε_co_ (f′_l_/f_co_)^0.7^ (11)

To evaluate the prediction capability of the proposed model, average absolute error (*AAE*) values of stress and strain functions are calculated for Points B and C (Table 8). For Point A, *AAE* values are not calculated since this point is defined to be on the conservative side. As seen in Table 8, the model predictions are good in agreement with the corresponding test data. 

## 6. Conclusions

Based on an experimental work on unconfined and CFRP confined circular HPFRCC specimens, the following conclusions can be drawn:

1. When confined with CFRP sheets, the axial strength and ultimate strain capacity of the circular HPFRCC specimens are enhanced significantly. The higher is the number of CFRP layers, the more remarkable is the increase in peak compressive strength and ultimate axial strain.
2. An average lateral FRP rupture strain of 0.006 and corresponding strain efficiency factor of 0.35 are obtained for HPFRCC confined by CFRP sheets. The values of these two key characteristics are observed to be less than FRP confined NSC and HSC. After the first ascending branch in the stress–strain curves, due to the different nature of HPFRCC in comparison with ordinary concrete, confinement pressure changed by local concentration of stresses resulting with lower hoop rupture strains and corresponding strain efficiency factors.
3. Investigation of five available models showed that only the model developed by Lim and Ozbakkaloglu [26] is in Category I in terms of axial strength and ultimate strain prediction performances. As seen Figure 5 and Figure 6, the predictions of the models are generally less accurate for axial strength and ultimate axial strain when stiffness of the FRP jacket is high. Obviously, there is a need for further research for different ranges of effective parameters, particularly for heavily confined HPFRCC.

## Figures and Tables

**Table 1:** Details of test specimens.

Designation	Number of Specimens	Number of CFRP Plies	FRP Volumetric Ratio (ρ_f_)
CC-C-0	3	Unconfined	-
CC-C-2	6	2	0.008
CC-C-4	3	4	0.018
CC-C-6	3	6	0.027
CC-C-8	4	8	0.035
CC-C-10	5	10	0.044

**Table 2:** Mix proportions of cementitious composite (kg/m^3^).

C	SF	S	STF	W	SP	Total
1000	250	815	78.5	124	125	2392.5

C: Cement (CEM I 42.5R); SF: Silica Fume (Norchem); S: Sand (0–0.5 mm); STF: Steel Fiber (Dramix, 60 mm, hooked-end); W: Water; SP: Superplastisizer (Chryso Optima 208).

**Table 3:** Mechanical properties of CFRP sheets.

Tensile Strength (MPa)	Tensile Elasticity Modulus (MPa)	Ultimate Tensile Deformation (%)	Effective Thickness (mm)
4200	240,000	1.8	0.166

**Table 4:** Test results.

Specimen	$f_{co}^{\prime }$	$\varepsilon_{co}$	$f_{cc}^{\prime }$	$f_{cu}^{\prime }$	Average Strength Loss	$\varepsilon_{cu}$	$\varepsilon_{h,\mathrm{rup}}$	$\varepsilon_{h,rup}$	$E_{c}$ (MPa)	$E_{c}$ (Average)	$f_{cc}^{\prime }/f_{co}^{\prime }$	$f_{cc}^{\prime }/f_{co}^{\prime }$	$\varepsilon_{cu}/\varepsilon_{co}$	$\varepsilon_{cu}/\varepsilon_{co}$	$k_{\varepsilon }$
	(MPa)	(%)	(MPa)	(MPa)		(%)	(%)	(Average)				(Average)		(Average)	(Average)
CC-C-2-1	102.7	0.300	126.6	108.9	0.36	0.907	0.521	0.577	36,695	39,524	1.23	1.38	3.02	2.10	0.32
CC-C-2-2	102.7	0.300	134.7	75.9		0.540 ^β^	0.601		39,658		1.31		1.80		
CC-C-2-3	102.7	0.300	137.4	80.3		0.805 ^β^	0.564		38,141		1.34		2.69		
CC-C-2-4	102.7	0.300	147.6	103.8		0.550 ^β^	0.597		36,489		1.44		1.83		
CC-C-2-5	102.7	0.300	150.2	100.2		0.410 ^β^	0.483		44,358		1.46		1.37		
CC-C-2-6	102.7	0.300	150.8	98.1		0.570 ^β^	0.701		41,806		1.47		1.90		
CC-C-4-1	102.7	0.300	149.3	136.7		1.062	0.636	0.559	35,850	34,765	1.45	1.55	3.54	4.32	0.31
CC-C-4-2 ^α^	102.7	0.300	172.1	-	0.21	1.722	-		34,259		1.68		5.74		
CC-C-4-3	102.7	0.300	155.7	124.2		1.106	0.482		34,187		1.52		3.69		
CC-C-6-1	102.7	0.300	159.9	-		1.571	0.576	0.632	37,784	38,951	1.56	1.61	5.24	4.91	0.35
CC-C-6-2	102.7	0.300	158.6	145.7	0.17	1.284	0.585		38,806		1.55		4.28		
CC-C-6-3	102.7	0.300	178.7	165.1		1.567	0.737		40,265		1.74		5.22		
CC-C-8-1	102.7	0.300	195.4	193.1		2.282	0.699	0.672	36,842	41,395	1.90	1.79	7.61	6.21	0.37
CC-C-8-2 ^α^	102.7	0.300	146.3	-	0.06	0.836	0.223		36,687		1.43		2.79		
CC-C-8-3	102.7	0.300	175.9	170.7		1.736	0.689		36,846		1.71		5.79		
CC-C-8-4	102.7	0.300	180.6	-		1.569	0.629		55,205		1.76		5.23		
CC-C-10-1 ^α^	102.7	0.300	150.7	-		0.448	0.301	0.677	34,092	37,146	1.47	1.90	1.49	6.80	0.38
CC-C-10-2	102.7	0.300	162.4	-		1.744	0.562		33,438		1.58		5.81		
CC-C-10-3	102.7	0.300	231.9	-	0.06	2.119	0.613		48,578		2.26		7.06		
CC-C-10-4 ^α^	102.7	0.300	168.6	-		2.244	0.267		31,468		1.64		7.48		
CC-C-10-5	102.7	0.300	218.5	-		2.054	0.856		38,154		2.13		6.85		

^α^: Due to problems with loading or instrumentation, excluded in calculations for average strain efficiency or strength/strain enhancement values, ^β^: Corresponding to 70% of the peak strength.

**Table 5:** Axial Strength and Ultimate Strain Prediction Expressions of Investigated Models.

Model/Strength Range	Strength Expression	Ultimate Strain Expression	Definition
[21,22]/25–82 MPa (Strength) 20–60 MPa (Strain)	$\frac{{f\prime }_{\mathrm{cc}}}{{f\prime }_{\mathrm{co}}}=1+4.2k_{\varepsilon }K_{s}$	$\varepsilon_{\mathrm{cu}}{=\varepsilon }_{\mathrm{co}}+0.17(0.6\varepsilon_{f}{-\varepsilon }_{\mathrm{co}})(\frac{E_{l}}{{f\prime }_{\mathrm{co}}}{)}^{0.65}$	$K_{s}=\frac{f^{\prime }{}_{l}}{{f\prime }_{\mathrm{co}}}$; ${f\prime }_{l}=\frac{{2E}_{f}\varepsilon_{f}t_{f}}{D}$; $E_{l}=\frac{{2E}_{f}t_{f}}{D}$; $k_{\varepsilon }=0.57$; $\varepsilon_{\mathrm{co}}=0.002$
[23]/20–200 MPa	$\frac{f_{\mathrm{cc}}^{\prime }}{f_{\mathrm{co}}^{\prime }}=1+\frac{k_{1}}{f_{\mathrm{co}}^{\prime }}f^{\prime }{}_{l}$	$\frac{\varepsilon_{\mathrm{cu}}}{\varepsilon_{\mathrm{co}}}=1+\frac{\sqrt{2}}{\varepsilon_{\mathrm{co}}}{\left(\frac{E_{l}}{{f\prime }_{\mathrm{co}}^{2}}\right)}^{2/3}{(\varepsilon }_{f}{-\upsilon }_{c}\varepsilon_{\mathrm{co}})$	$k_{1}=\frac{9.5}{{f\prime }_{\mathrm{co}}{}^{1/4}}$; $\upsilon_{c}=0.2$; $k_{\varepsilon }=1$; $\varepsilon_{\mathrm{co}}=0.002$
[24]/up to 200 MPa	$\frac{f_{\mathrm{cc}}^{\prime }}{f_{\mathrm{co}}^{\prime }}=1+3.55(\frac{{f\prime }_{l}}{f_{\mathrm{co}}}{)}^{0.15}$	$\frac{\varepsilon_{\mathrm{cu}}}{\varepsilon_{\mathrm{co}}}=2+23(\frac{{f\prime }_{l}}{f_{\mathrm{co}}^{\prime }})$	$k_{\varepsilon }=0.50$
[25]/18–55 MPa	$\frac{{f\prime }_{\mathrm{cc}}}{{f\prime }_{\mathrm{co}}}=1+2.2(\frac{{f\prime }_{l}}{{f\prime }_{\mathrm{co}}}{)}^{0.94}$	$\frac{\varepsilon_{\mathrm{cu}}}{\varepsilon_{\mathrm{co}}}=1.75+12(\frac{{f\prime }_{l}}{{f\prime }_{\mathrm{co}}}{)}^{0.75}{\left(\frac{f_{30}}{{f\prime }_{\mathrm{co}}}\right)}^{0.62}$	$k_{\varepsilon }=1$; $\varepsilon_{\mathrm{co}}=0.0030$
[26]/6–170 MPa	$f_{\mathrm{cc}}^{\prime }{=c}_{1}f_{\mathrm{co}}^{\prime }+3.52{(f\prime }_{l}{-f}_{1o})$	$\varepsilon_{\mathrm{cu}}{=c}_{2}\varepsilon_{\mathrm{co}}+0.272(E_{l}{/f}_{\mathrm{co}}^{\prime })\varepsilon_{h,\mathrm{rup}}{}^{1.35}$ $\varepsilon_{\mathrm{co}}=(-0.67{f\prime }_{\mathrm{co}}{}^{2}+29.9{f\prime }_{\mathrm{co}}+1053)10^{-6}$	$c_{1}=1+0.0058E_{l}{/f\prime }_{\mathrm{co}}{;f}_{\mathrm{lo}}{=E}_{l}\varepsilon_{\mathrm{co}}(0.43+0.009E_{l}{/f\prime }_{\mathrm{co}}{)(E}_{l}{³K}_{1o})$ $c_{1}{=(E}_{l}{/f\prime }_{\mathrm{co}}{}^{1.6}{)}^{0.2}{;f}_{\mathrm{lo}}{=24E}_{l}\varepsilon_{\mathrm{co}}{{(f\prime }_{\mathrm{co}}{/E}_{l}{}^{1.6})}^{0.4}{(E}_{l}{<}_{1o})$ $c_{2}=2-(\frac{{f\prime }_{\mathrm{co}}-20}{100})$; $k_{\varepsilon }=0.9-2.3{f\prime }_{\mathrm{co}}10^{-3}-0.75E_{f}10^{-6}$; $\varepsilon_{\mathrm{co}}=0.0034$

$f\prime_{l}=$ Lateral confinement pressure at ultimate; $\upsilon_{c}=$ Poisson ratio; $f_{30}=$ 30 Mpa.

**Table 6:** Statistics for models.

Models	f’_cc_/f’_co_		Ɛ_cu_/Ɛ_co_	
	*AAE*	*SD*	*AAE*	*SD*
[21,22]	0.34	0.28	0.55	0.35
[23]	0.50	0.39	0.32	0.25
[24]	0.12	0.15	0.62	0.36
[25]	0.30	0.24	0.12	0.23
[26]	0.14	0.17	0.10	0.11

**Table 7:** Model performances.

Models	Category I		Category II		Category III	
	Strength	Strain	Strength	Strain	Strength	Strain
[21,22]					x	x
[23]					x	x
[24]	x					x
[25]		x	x			
[26]	x	x				

**Table 8:** *AAE* values of the proposed model.

Point	Stress	Strain
B	0.03	0.04
C	0.11	0.06
